# Supplementary material for: Cuscuta Species Identification Based on the Morphology of Reproductive Organs and Complete Chloroplast Genome Sequences
Source: Int J Mol Sci. 2019 Jun 3;20(11):2726. doi: 10.3390/ijms20112726 (PMC6600609; doi:10.3390/ijms20112726)
Supplement: Supplementary file 1 [file ijms-20-02726-s001.pdf]

Supplementary Materials: *Cuscuta* species identification based on the morphology of reproductive organs and complete chloroplast genome sequences

Inkyu Park†, Jun-Ho Song†, Sungyu Yang, Wook jin Kim, Goya Choi, Byeong Cheol Moon*

†These authors contributed equally to this work.

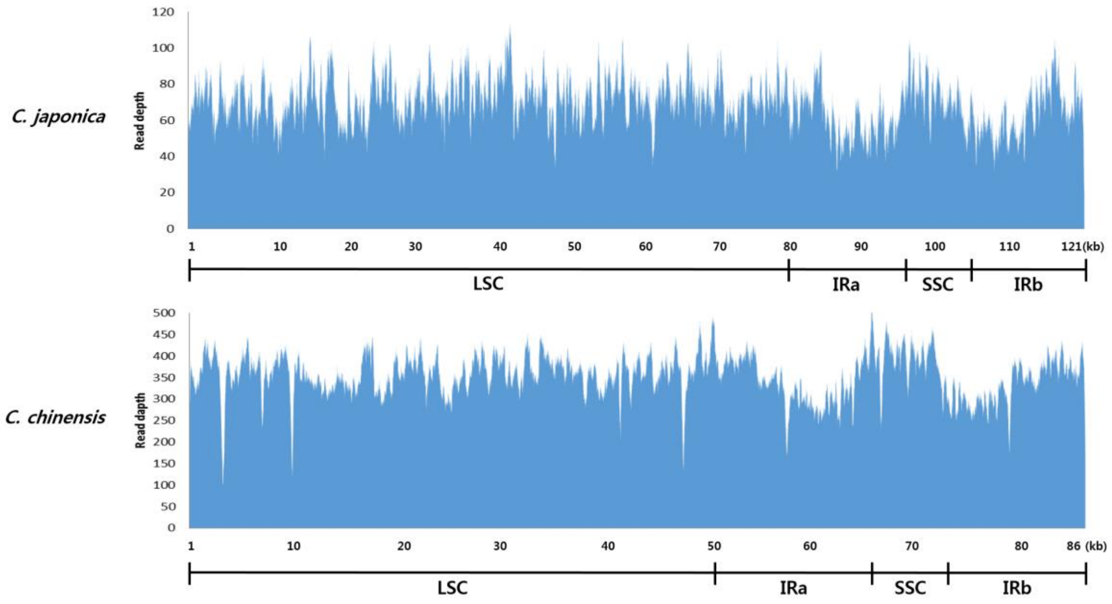

Figure S1: Distribution of paired-end reads mapped onto the complete chloroplast genomes of two *Cuscuta* species, *C. japonica* and *C. chinensis*. LSC, large single copy region; SSC, small single copy region; IRa, inverted repeat a; IRb, inverted repeat b.

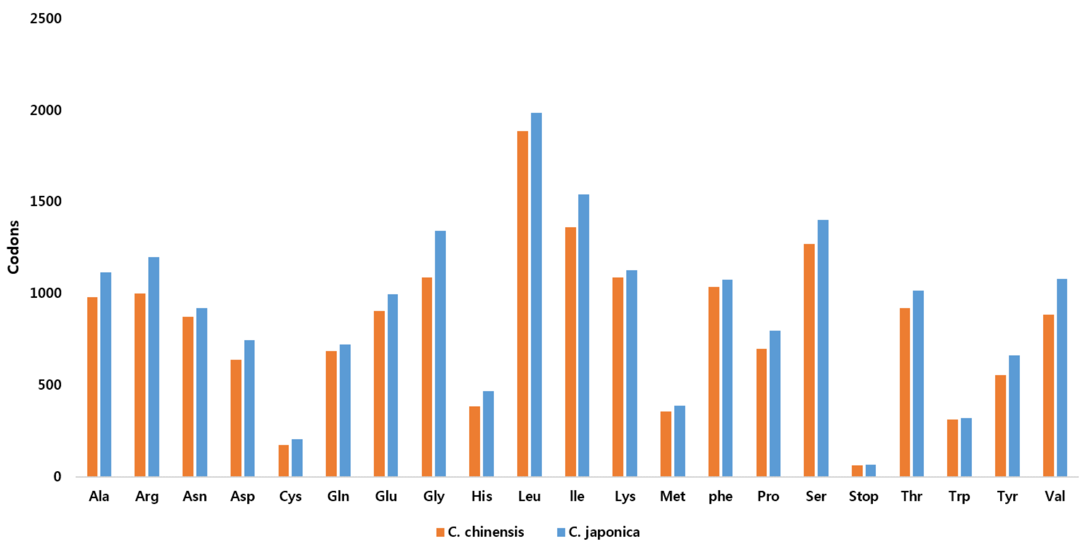

Figure S2: Frequencies of amino acids in protein-coding regions in chloroplast genomes of *C. japonica* and *C. chinensis*.

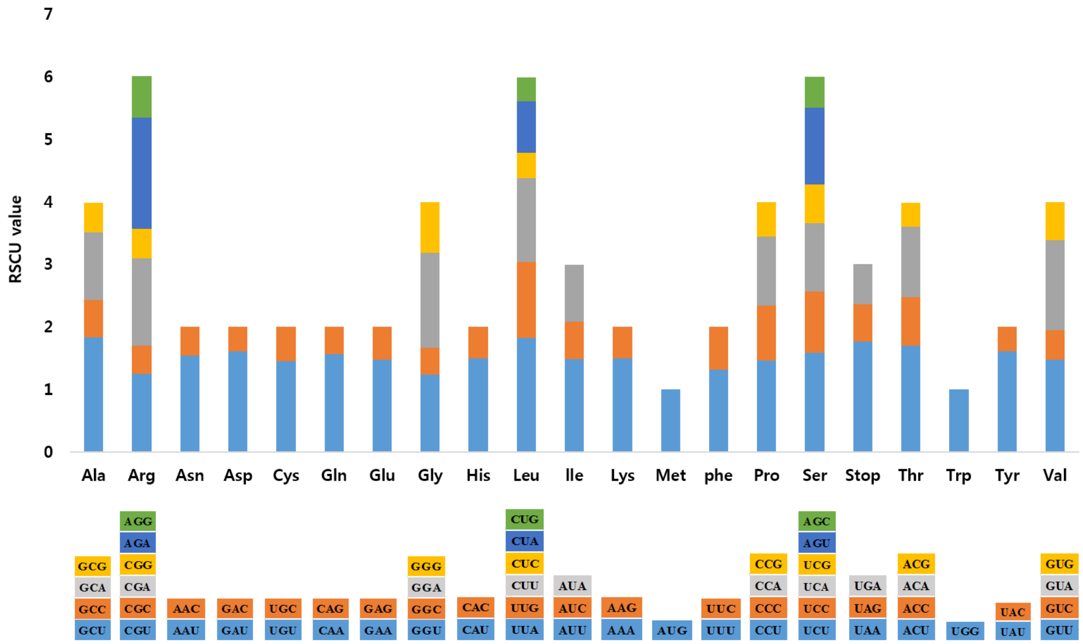

Figure S3: Relative synonymous codon usage (RSCU) values of 20 amino acids and stop codon in 65 and 58 protein-coding genes in chloroplast genomes of *C. japonica* and *C. chinensis*, respectively.

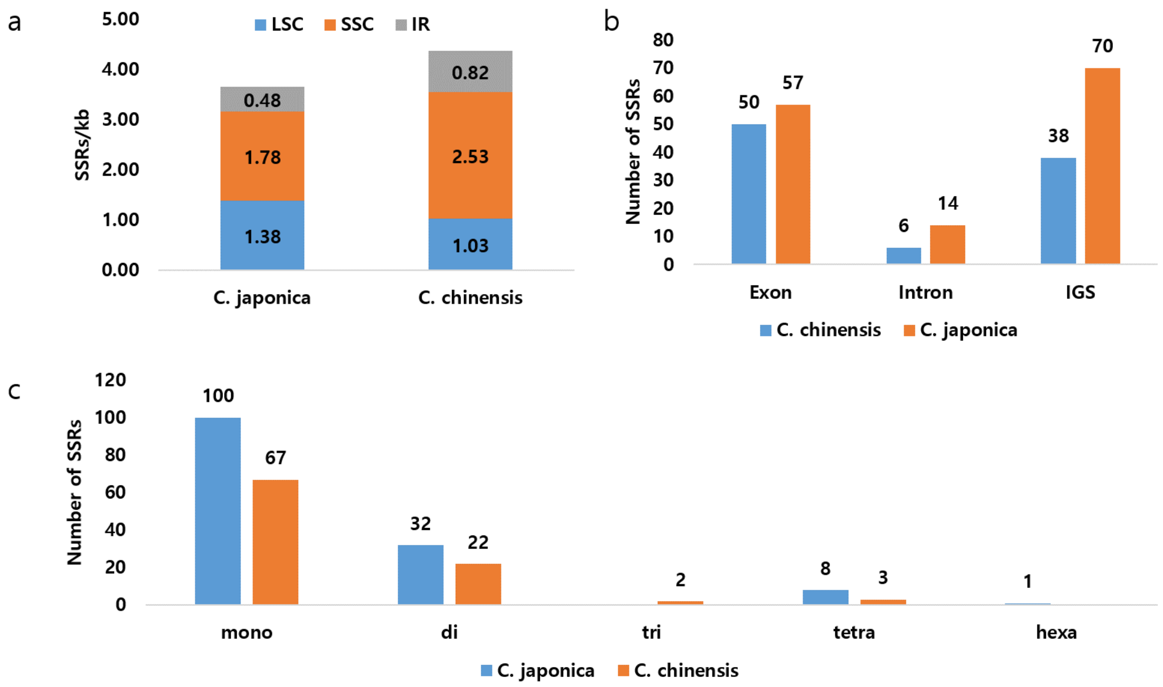

Figure S4: Distribution of simple sequence repeats (SSRs) in chloroplast genomes of *C. japonica* and *C. chinensis*. (**a**) Number of SSRs per unit length. (**b**) Distribution of SSRs in exons, introns and intergenic spacer (IGS) regions. (**c**) Distribution of different types of SSRs.

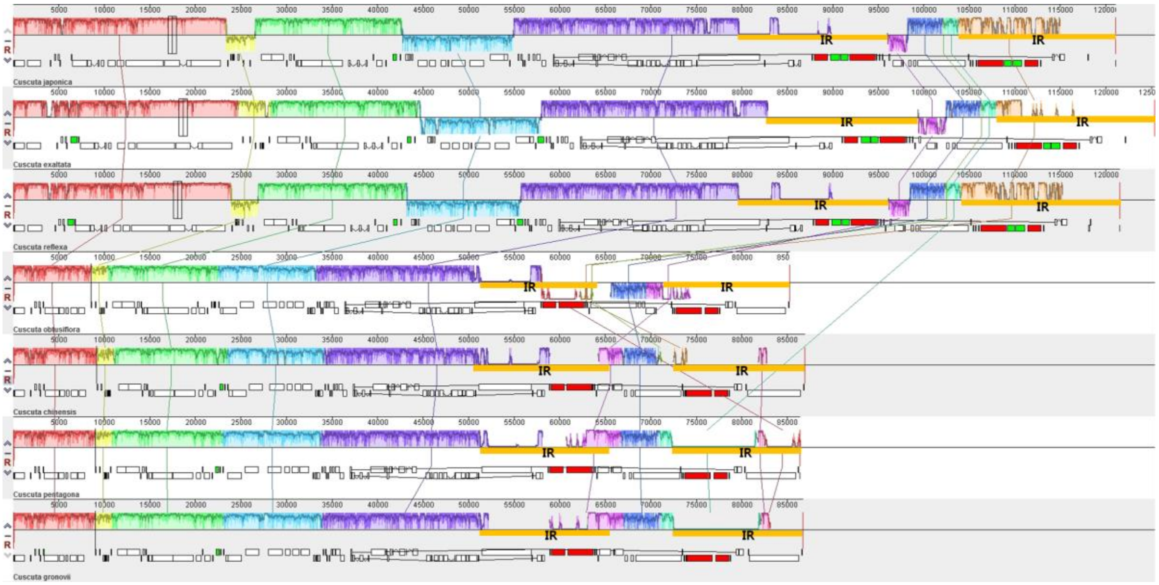

Figure S5: Comparison of complete chloroplast genomes of seven *Cuscuta* species using the MAUVE algorithm. Local collinear blocks coloured in this figure indicate syntenic regions, while histograms within each block represent the degree of sequence similarity.

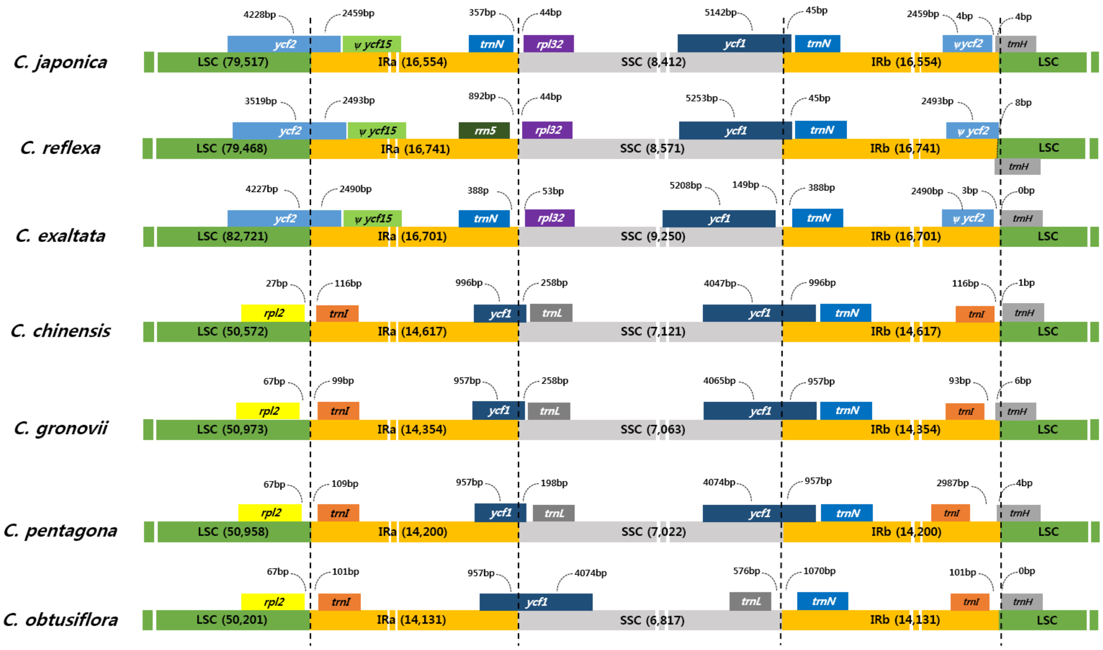

Figure S6: Comparison of the position of large single copy (LSC), inverted repeat (IR) and small single copy (SSC) junctions among chloroplast genomes of seven *Cuscuta* species.

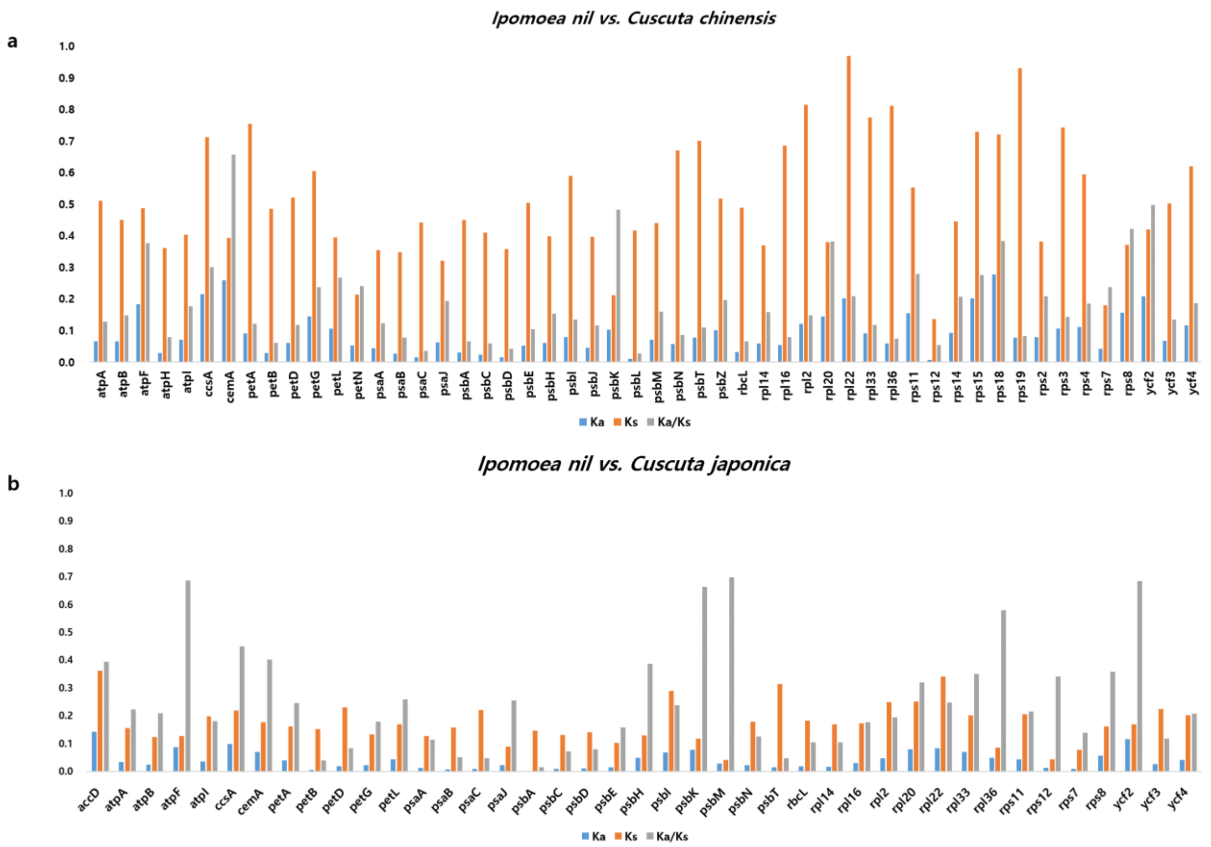

Figure S7: Comparison of non-synonymous to synonymous substitution ratios (Ka/Ks) of 50 conserved protein-coding genes among the chloroplast genomes of *C. chinensis*, *C. japonica* and *Ipomoea nil*. (a) Comparison between *Ipomoea nil* and *C. chinensis*. (b) Comparison between *I. nil* and *C. japonica*. Blue and orange bars indicate Ka and Ks values, respectively, while grey bars indicate Ka/Ks ratios. Genes with Ka or Ks = 0 are not shown.

Table S1: Morphological and micromorphological characteristics of reproductive organs of *Cuscuta japonica* and *Cuscuta chinensis*.

	C. japonica	***C. chinensis***
Style number	One	Two (equal or unequal)
Style surface cell type	Striate	Striate
Stigma shape	Cylindrical, ellipsoid	Capitate
Stigma surface cell type	Papillate	Papillate
Ovary surface cell type	Papillate	Papillate
Fimbriae of infrastaminal scale (IFS) type	Glandular cells	Secretory cells
Seed shape	Pyriform to oblate	Globose to obovoid
Testa ornamentation	Rugulated (puzzle-like arrangement)	Reticulate (net-like arrangement)

Table S2: Raw read and assembly information of two *Cuscuta* chloroplast genomes.

	C. japonica	***C. chinensis***
Input reads	7,089,862	7,307,224
Trimmed reads	6,168,760	6,258,253
Total raw bases (bp)	2,020,636,368	2,177,913,092
Trimmed bases (bp)	1,528,063,366	1,530,019,927
Aligned reads	34,681	126,584
Coverage (×)	69.58	354.8
Chloroplast genome length (bp)	121,037	86,927

Table S3: Primers used for PCR-based sequence validation of *C. japonica* and *C. chinensis* chloroplast junctions.

Primer name	Primer sequence (5′→3′)		Junction†
	C. japonica	*C. chinensis*	
LI_F	TTTGTTTCGCGGGCGTTTAG	ACAATGGCGCTTTCCAGAGA	LSC/IRa
LI_R	TGCTCCCGGTTGTTCAATCA	ATTCCTACTGGATGCACGCT	
IS_F	TCGTAGTGGCTCCCTTCCATA	CGGGCGTAGCAAAAGAAGAG	IRa/SSC
IS_R	AAATTCCCGGTCAAAAGGGAT	CAATTTCACCATAGCGGCGG	
SI_F	CGGGCTAGCGTTGAATTTGG	CGGGCGTAGCAAAAGAAGAG	SSC/IRb
SI_R	CTTCGTAGTGGCTCCCTTCC	CAATTTCACCATAGCGGCGG	
IL_F	TGCTCCCGGTTGTTCAATCA	CCCTTACCTATTCCGACGCA	IRb/LSC
IL_R	AGGCGTAGCTGGTGTATTCG	CCAATTTCACCATAGCGGCG	

†LSC, large single copy region; SSC, small single copy region; IRa, inverted repeat a; IRb, inverted repeat b.

Table S4: Results of PCR-based sequencing of chloroplast junctions.

Species	Junction†	PCR product size (bp)	Start position (bp)	End position (bp)	Identity (%)
C. japonica	LSC/IRa	581	79,226	79,765	100
	IRa/SSC	292	95,949	96,240	100
	SSC/IRb	566	104,041	104,606	100
	IRb/LSC	375	120,748	85	100
C. chinensis	LSC/IRa	261	50,444	50,704	100
	IRa/SSC	747	65,038	65,784	100
	SSC/IRb	228	72,311	72,538	100
	IRb/LSC	747	86,213	32	100

†LSC, large single copy region; SSC, small single copy region; IRa, inverted repeat a; IRb, inverted repeat b.

Table S5: Genes present in the chloroplast genome of *C. japonica*.

Gene group	Gene name
Photosystem I	*psaA, B, C, I, J, ycf3*[†], *ycf4*
Photosystem II	*psbA, B, C, D, E, F, H, I, J, K, L, M, N, T, Z*
Cytochrome b6/f	*petA, B*[§], *D*[§], *G, L, N*
ATP synthase	*atpA, B, E, F*[§], *H, I*
Rubisco	*rbcL*
Large subunit ribosomal proteins	*rpl2, 14, 16*[§], *20, 22, 32, 33, 36*
Small subunit ribosomal proteins	*rps2, 3, 4, 7*[¶], *8, 11, 12*[†, ¶, ¥], *14, 15, 18, 19*
RNA polymerase	*rpoA, B, C1*[§], *C2*
Protein-coding gene with unknown function	*ycf1, ycf2*
Other genes	*accD, ccsA, cemA, clpP*[†], *matK*
Ribosomal RNA (rRNA) genes	*rrn16*[¶], *23*[¶], *4.5*[¶], *5*[¶]
Transfer RNA (tRNA) genes	*trnA-UGC*[§, ¶], *trnC-GCA, trnD-GUC, trnE-UUC, trnF-GAA, trnfM-CAU, trnG-GCC, trnH-GUG, trnI-CAU, trnI-GAU*[§, ¶], *trnL-UAA, trnL-UAG, trnL-CAA*[¶], *trnM-CAU, trnN-GUU*[¶], *trnP-UGG, trnQ-UUG, trnR-ACG*[¶], *trnR-UCU, trnS-GCU, trnS-GGA, trnS-UGA, trnT-GGU, trnT-UGU, trnV-GAC*[¶], *trnW-CCA, trnY-GUA*

[§]Genes containing a single intron. [†]Genes containing two introns. [¶]Genes with two copies in inverted repeats (IRs). [¥]Trans-splicing gene.

Table S6: Genes present in the chloroplast genome of *C. chinensis*.

Gene group	Gene name
Photosystem I	*psaA, B, C, J, ycf3, ycf4*
Photosystem II	*psbA, B, C, D, E, F, H, I, J, K, L, M, N, T, Z*
Cytochrome b6/f	*petA, B*§, *D*§, *G, L, N*
ATP synthase	*atpA, B, E, F, H, I*
Rubisco	*rbcL*
Large subunit ribosomal proteins	*rpl2*§, ¶, *14, 16*§, *20, 22, 33, 36*
Small subunit ribosomal proteins	*rps2, 3, 4, 7*¶, *8, 11, 12*†, ¶, ¥, *14, 15, 18, 19*
Protein-coding gene with unknown function	*ycf1*¶, *2*¶
Other genes	*accD, ccsA, cemA, clpP*†
rRNA genes	*rrn16*¶, *23*¶, *4.5*¶, *5*¶
tRNA genes	*trnC-GCA, trnD-GUC, trnE-UUC, trnF-GAA, trnfM-CAU, trnG-GCC, trnH-GUG, trnI-CAU*¶, *trnL-UAA, trnL-UAG, trnM-CAU, trnN-GUU*¶, *trnP-UGG, trnQ-UUG, trnR-UCU, trnS-GCU, trnS-GGA, trnS-UGA, trnT-UGU, trnV-GAC, trnV-UAC, trnW-CCA, trnY-GUA*

§Genes containing a single intron. †Genes containing two introns. ¶Genes with two copies in inverted repeats (IRs). ¥Trans-splicing gene.

Table S7: Genes lost from the chloroplast genomes of *C. japonica* and *C. chinensis*.

Gene group	Gene name	
	C. japonica	***C. chinensis***
Photosystem I	-	*psa I*
NADH oxidoreductase	*ndhA, B, C, D, E, F, G, H, I, J, K*	*ndhA, B, C, D, E, F, G, H, I, J, K*
Large subunit ribosomal proteins	*rpl23*	*rpl23, 32*
Small subunit ribosomal proteins	*rps16*	*rps16*
RNA polymerase	-	*rpoA, B, C1, C2*
Other genes	-	*matK*
tRNA genes	*trnG-UCC, trnK-UUU, trnL-CAA, trnV-UAC*	*trnA-UGC, trnG-UCC, trnI-GAU, trnK-UUU, trnL-CAA, trnR-ACG, trnT-GGU, trnV-UAC*

Table S8: Lengths of exons and introns of genes in chloroplast genomes of *C. japonica* and *C. chinensis*.

	Gene name	Location†	Exon I§	Intron I§	Exon II§	Intron II	Exon III
1	*atpF*	LSC	145	707	410		
2	*rpoC1*	LSC	435	811	1623		
3	*ycf3*	LSC	126	740	228	782	153
4	*clpP*	LSC	71 (71)	732 (586)	292 (292)	661	243
5	*petB*	LSC	6 (6)	759 (662)	642 (642)		
6	*petD*	LSC	8 (8)	695 (692)	475 (475)		
7	*rpl16*	LSC	9 (9)	953 (885)	408 (405)		
8	*trnI-GAU*	IR	37	951	35		
9	*trnA-UGC*	IR	38	782	35		

†LSC, large single copy region; SSC, small single copy region; IR, inverted repeat region. §Numbers in parentheses represent the length of corresponding sequences in *C. chinensis*.

Table S9: Tandem repeats detected in *C. japonica* and *C. chinensis* chloroplast genomes.

C. japonica	**Position†**	**Repeat unit length (bp)**	**Repeat unit sequence**	**Repeat number**	**Location§**
1	IGS (*matK*, *trnQ-UUG*)	38	TCATACCTGATCTCATAAC	2	LSC
2	IGS (*trnS-GCU*, *trnR-UCU*)	80	ATAATAGATTAATATATTAA	4	LSC
3	IGS (*atpF*, *atpH*)	38	TTATAGATAGACTAAAAAA	2	LSC
4	IGS (*atpH*, *atpI*)	30	AATATTATATTATTG	2	LSC
5	Exon (*accD*)	30	ACATAATATAAGAAA	2	LSC
6	Exon (*rps18*)	42	TAAATCCAAGCGACCTTTTAT	2	LSC
7	IGS (*clpP*, *psbB*)	24	CATAGTATAAGT	2	LSC
8	Exon (*ycf2*)	24	TTTCAAGTTATG	2	LSC
9	IGS (*trnN-GUU*, *rpl32*)	45	TATATCTTATTAGAG	3	IRa/SSC junction
10	IGS (*ccsA*, *trnL-UAG*)	30	TATGATATATAAATA	2	SSC
11	IGS (*rps15*, *ycf1*)	30	TATACATTCGTACTA	2	SSC
12	Exon (*ycf1*)	240	TTTTTTGTTTACTATCTATTTCAC	10	SSC
13	IGS (*ycf1*, *trnN-GUU*)	45	ATATACTCTAATAAG	3	SSC/IRb junction
C. chinensis					
1	IGS (*trnQ-UUG*, *psbK*)	44	TATTATAATATTTAATATTATT	2	LSC
2	IGS (*trnE-UUC*, *psbD*)	40	TATTTAATAAAATAATATAA	2	LSC
3	IGS (*psbN*, *psbH*)	28	ATATTATTATATAA	2	LSC
4	Exon (*ycf2*)	42	TCAATTACGTGTTTATATTCT	2	IR
5	Exon (*ycf2*)	42	AAGAATAAAAAAACGTAATTG	2	IR

†IGS, intergenic spacer region. §LSC, large single copy region; SSC, small single copy region; IRa, inverted repeat a; IRb, inverted repeat b.

Table S10: Accession numbers of chloroplast genomes used for phylogenetic analysis.

Family	Taxon	GenBank accession number
Convolvulaceae	*Ipomoea purpurea*	EU118126.1
	Ipomoea obscura	KF242499.1
	Ipomoea minutiflora	KF242498.1
	Ipomoea tricolor	KF242495.1
	Ipomoea nil	KF242487.1
	Ipomoea murucoides	KF242486.1
	Ipomoea involucrata	KF242485.1
	Ipomoea hederifolia	KF242484.1
	Ipomoea batatas	KF242475.1
	Cressa cretica	NC035516.1
	Cuscuta exaltata	NC009963.1
	Cuscuta reflexa	NC009766.1
	Cuscuta obtusiflora	NC009949.1
	Cuscuta gronovii	NC009765.1
	Cuscuta pentagona	NC039759.1
	Cuscuta japonica	MH780080
	Cuscuta chinensis	MH780079
Solanaceae	*Nicotiana tabacum*	NC_001879.2
	Capsicum annuum var. *glabriusculum*	KJ619462.1

Table S11: List of *C. japonica* and *C. chinensis* specimens examined in this study.

C. japonica	Collection site	Collection date	Collector and voucher number	Herbarium †	Study§
1	Jeju, Jeju-si, Gujwa-eup	31st August 2016	Moon & Yang, KIOM201601017941	KIOM	M, C
2	Jeju, Jeju-si, Hallim-eup	29th August 2016	Moon & Yang, KIOM201601017916	KIOM	M
3	Chungbuk, Danyang-gun	28th July 2016	Moon & Yang, KIOM201701018784	KIOM	M
4	Chungbuk, Chungju-si	27th August 2014	Moon, KIOM201501013896	KIOM	M
5	Chungbuk, Goesan-gun	31st October 2016	Ko, HNU-2016-1368	KB	M
6	Gangwon, Jeongseon-gun	13th October 2016	Nam et al., HNU-2016-1368	KB	M
C. chinensis					
1	Jeju, Jeju-si, Hallim-eup	29th August 2016	Moon & Yang, KIOM201601017927	KIOM	M, C
2	Jeju, Jeju-si, Hallim-eup	29th August 2016	Moon & Yang, KIOM201601017928	KIOM	M
3	Jeju, Jeju-si, Hallim-eup	29th August 2016	Moon & Yang, KIOM201601017929	KIOM	M
4	Gangwon, Samcheok-si	19th August 2011	Kim et al., SHY2-748	KB	M

†KB, National Institute of Biological Resources; KIOM, Korea Institute of Oriental Medicine; Herbarium acronyms follow Index Herbariorum [continuously updated] http://sweetgum.nybg.org/science/ih/.

§M, morphological and micromorphological studies; C, chloroplast genome sequencing.

Table S12: Best-fitting substitution models selection using jModelTest

	Model	f(a)	f(c)	f(g)	f(t)	kappa	titv	Ra	Rb	Rc	Rd	Re	Rf	pInv	gamma
AIC	GTR+I+G	0.3	0.18	0.21	0.31	0	0	1.695	2.013	0.254	0.849	2.137	1	0.27	0.87

Model	-lnL*	K	AIC	delta	weight	cumWeight
GTR+I+G	128183.1683	46	256458.3365	0	0.997641	0.997641
GTR+G	128190.2153	45	256470.4307	12.09412	0.002359	1
GTR+I	128265.7386	45	256621.4773	163.14072	3.74E-36	1
HKY+I+G	129136.7981	42	258357.5963	1899.25974	0.00E+00	1
HKY+G	129143.9543	41	258369.9087	1911.57214	0.00E+00	1
HKY+I	129207.9256	41	258497.8512	2039.5147	0.00E+00	1
SYM+I+G	129477.1116	43	259040.2231	2581.88656	0.00E+00	1
SYM+G	129484.9678	42	259053.9356	2595.59908	0.00E+00	1
SYM+I	129550.8414	42	259185.6827	2727.3462	0.00E+00	1
GTR	129681.2137	44	259450.4273	2992.09078	0.00E+00	1
K80+I+G	130110.5561	39	260299.1122	3840.77564	0.00E+00	1
K80+G	130118.5079	38	260313.0158	3854.6793	0.00E+00	1
K80+I	130182.1891	38	260440.3782	3982.0417	0.00E+00	1
F81+I+G	130210.5895	41	260503.179	4044.84248	0.00E+00	1
F81+G	130217.2105	40	260514.4209	4056.0844	0.00E+00	1
F81+I	130277.8176	40	260635.6352	4177.29868	0.00E+00	1
HKY	130652.8223	40	261385.6446	4927.30808	0.00E+00	1
SYM	131019.6459	41	262121.2917	5662.9552	0.00E+00	1
JC+I+G	131090.6796	38	262257.3592	5799.02268	0.00E+00	1
JC+G	131097.8017	37	262269.6033	5811.2668	0.00E+00	1
JC+I	131159.0792	37	262392.1584	5933.82184	0.00E+00	1
K80	131678.6046	37	263431.2092	6972.87262	0.00E+00	1
F81	131698.7243	39	263475.4485	7017.11198	0.00E+00	1
JC	132612.4124	36	265296.8248	8838.48828	0.00E+00	1

*-lnL: negative log likelihod; K: number of estimated parameters; AIC: Akaike Information Criterion; delta: AIC difference; weight: AIC weight; cumWeight: cumulative AIC weight
